# Effect of nursing care hours on the outcomes of Intensive Care assistance

**DOI:** 10.1371/journal.pone.0188241

**Published:** 2017-11-30

**Authors:** Tatiana do Altíssimo Nogueira, Mayra Gonçalves Menegueti, Gleice da Silva Castro Perdoná, Maria Auxiliadora-Martins, Fernanda Maria Togeiro Fugulin, Ana Maria Laus

**Affiliations:** 1 Department of Fundamental Nursing, University of São Paulo at Ribeirão Preto College of Nursing, Ribeirão Preto, São Paulo, Brazil; 2 Department of Social Medicine, University of São Paulo at Ribeirão Preto Faculty of Medicine, Ribeirão Preto, São Paulo, Brazil; 3 Department of Surgery and Anatomy, Division of Intensive Care University of São Paulo at Ribeirão Preto Faculty of Medicine, Ribeirão Preto, São Paulo, Brazil; 4 Department of Professional Orientation, University of São Paulo School of Nursing, São Paulo, São Paulo, Brazil; Yokohama City University, JAPAN

## Abstract

**Objectives:**

To correlate the average number of nursing care hours dedicated to Intensive Care Unit (ICU) patients with nursing care indicators.

**Method:**

Transverse, descriptive study conducted between 2011 and 2013. Data were obtained from the electronic records system and from the nursing staff daily schedule. Generalized Linear Models were used for analysis.

**Results:**

A total of 1,717 patients were included in the study. The average NAS (Nursing Activities Score) value was 54.87. The average ratio between the number of nursing care hours provided to the patient and the number of nursing care hours required by the patient (hours ratio) was 0.87. Analysis of the correlation between nursing care indicators and the hours ratio showed that the indicators phlebitis and ventilator-associated pneumonia significantly correlated with hours ratio; that is, the higher the hours ratio, the lower the incidence of phlebitis and ventilator-associated pneumonia.

**Conclusion:**

The number of nursing care hours directly impacts patient outcomes, which makes adjustment of nurse staffing levels essential.

## Introduction

Alterations in healthcare services due to changing epidemiological profile, population aging, technological advances, and more demanding healthcare users require process optimization, increased productivity, improved patient care quality, and reduced costs [1].

Nursing care is essential to ensure safe healthcare delivery. Nursing care quality encompasses variables such as workload, work environment, patient severity, nursing staff qualification, and cost-effectiveness as measured by patient clinical outcomes [2]. Adequate nurse staffing has become a fundamental strategy in healthcare quality management because it can impact clinical indicators.

A study has shown that mortality decreases at lower patient-to-nurse ratios [3]. Addition of a patient to the nursing workload increases emotional distress, reduces job satisfaction, and raises mortality [4–6]. In this sense, re-dimensioning of nurse staffing affects healthcare indicators like hospital infections [7,8] and accidental extubation [9].

The Intensive Care Unit (ICU) is the hospital section that is the most vulnerable to adverse events. A study has pointed out that over 20% of ICU patients have experienced some kind of adverse event [10]. The medical literature has suggested the use of various healthcare indicators to evaluate healthcare quality; these indicators include medication-related adverse events, pressure ulcer, fall, pneumonia, and hospital-acquired infections, among others [11,12]. It is imperative that continuous organizational, managerial, and operational assessment (including human resources) of the ICU be conducted in order to ensure better nursing care practices, continuous staff training, and care delivery excellence.

Despite the many studies on how the number of nursing care hours impacts the outcome of ICU patients, results have been inconclusive. In most cases, the aforementioned relationship has been analyzed by considering either the number of nursing care hours provided to the patient or the number of nursing care hours required by the patient. However, if we consider the possibility of comparing or establishing a relationship (ratio) between these numbers of nursing care hours, we could obtain a more precise value that would allow us to demonstrate that adequate nurse staffing can positively impact the outcomes of the care offered to ICU patients and their relatives.

The present investigation aimed to analyze the ratio between the number of nursing care hours provided to the patient and the number of nursing care hours required by the patient (hours ratio) in an ICU and to correlate this ratio with the care indicators evaluated in the unit.

## Method

This is a transverse, descriptive, and quantitative study conducted at the medium-sized ICU (eight hospital beds) of a private hospital in a city of the state of São Paulo, Brazil. The hospital assists private patients and health plan users. The ICU implemented an electronic records system to monitor and manage patient care and safety indicators and to file information regarding patient stay in the ICU. The present study was approved by the Research Ethics Committee of Ribeirão Preto College of Nurse, University of São Paulo (EERP/USP), Ribeirão Preto/SP, Brazil (Protocol No. 470.342/2013), waiving the need for consent from study participants. Data was accessed anonymously.

The Nursing Activities Score (NAS) measures the workload of assisting nurses prospectively, in all shifts. The electronic records system adds up the scores obtained after the nursing staff members fill in the NAS form, to calculate the average daily and monthly values of ICU patients. The care indicators phlebitis, accidental or unplanned extubation of the orotracheal cannula, pressure ulcer, and unplanned removal of the feeding tube were selected from the Handbook of Nursing Care Indicators of the Hospital Management Support Hub (HMSH) of the Program of Hospital Quality Assurance (HQA) [13]. Indicators related to the incidence of nosocomial infections [ventilation-associated pneumonia (VAP) and urinary tract infection (UTI)] were also considered because they reflected a specific feature of the ICU.

Data were collected from the database stored in the electronic records system of the surveyed ICU. The study population consisted of all the patients admitted to the ICU between 01 January 2011 and 31 December 2013 for at least 24 h. The researcher was a member of the nursing staff of the ICU. One of this researcher’s attributions was to insert data into the electronic records system after daily assessment of the ICU patients, which made all the information and monitoring of clinical indicators more reliable.

Quantitative data were based on the ICU nursing staff daily and monthly schedules.

The following equation was used to calculate the average number of nursing care hours required by the patient [14]:

h=NAS×24÷100

Legend
h = average number of nursing care hours required by the patient
NAS = average NAS value
24/100 = relationship corresponding to 24 h per 100 NAS points

To obtain the average number of nursing care hours provided to the patient on the basis of the nursing staff schedule, the following equation was employed, [15]:

hk=qk×t÷n

Legend
h*k* = average number of hours provided to each patient by professionals belonging to category *k* (nurse or nurse technician)
q*k* = average number of professionals belonging to category *k*
t = workload
n = average number of patients per day

The ratio between the number of nursing care hours provided to the patient and the number of nursing care hours required by the patient was also calculated (hours ratio). Hours ratios closer to one meant a smaller difference between the number of provided and required nursing care hours.

Care indicators were calculated by using the equations recommended in the Handbook of Nursing Care Indicators. The values of these indicators were related to the incidence of nosocomial infections (VAP and UTI) established by *NNISS* (National Nosocomial Infections Surveillance System)[16].

The data were transferred to a Microsoft Excel spreadsheet and were descriptively analyzed by using the program SPSS (*Statistical Package for the Social Sciences*).

To analyze the data, Generalized Linear Models were applied. A binomial distribution of responses was considered for the variable phlebitis, VAP, and UTI. The asymmetric distribution of the variables phlebitis, accidental or unplanned extubation of the orotracheal cannula, VAP, UTI, pressure ulcer, and unplanned removal of the feeding tube was considered as gamma distribution of responses. The level of significance was 5% (α = 0.05).

## Results

The clinical and demographic profile of the patients admitted to the studied ICU indicated that the Unit predominantly admitted clinical patients (70%) with respiratory and cardiovascular diseases. Fifty percent of the patients came from the emergency room; the average ICU stay was 3.85 days; 50.8% of the patients were male; and 61% of the patients were aged at least 65 years. The average severity index SAPS 3 was 48.60 points for the patients evaluated along the study period.

The total number of nursing care hours provided to the patient was 13.28 h (maximum value) and 10.23 h (minimum). Considering professional categories, between 77 and 70% of these hours corresponded to the work of nurse technicians, and between 23 and 30% of these hours referred to the work of nurses.

The average number of nursing care hours required by the patient was 13.58 h (maximum value) and 12.42 (minimum value), as calculated according to NAS.

**Fig 1:**
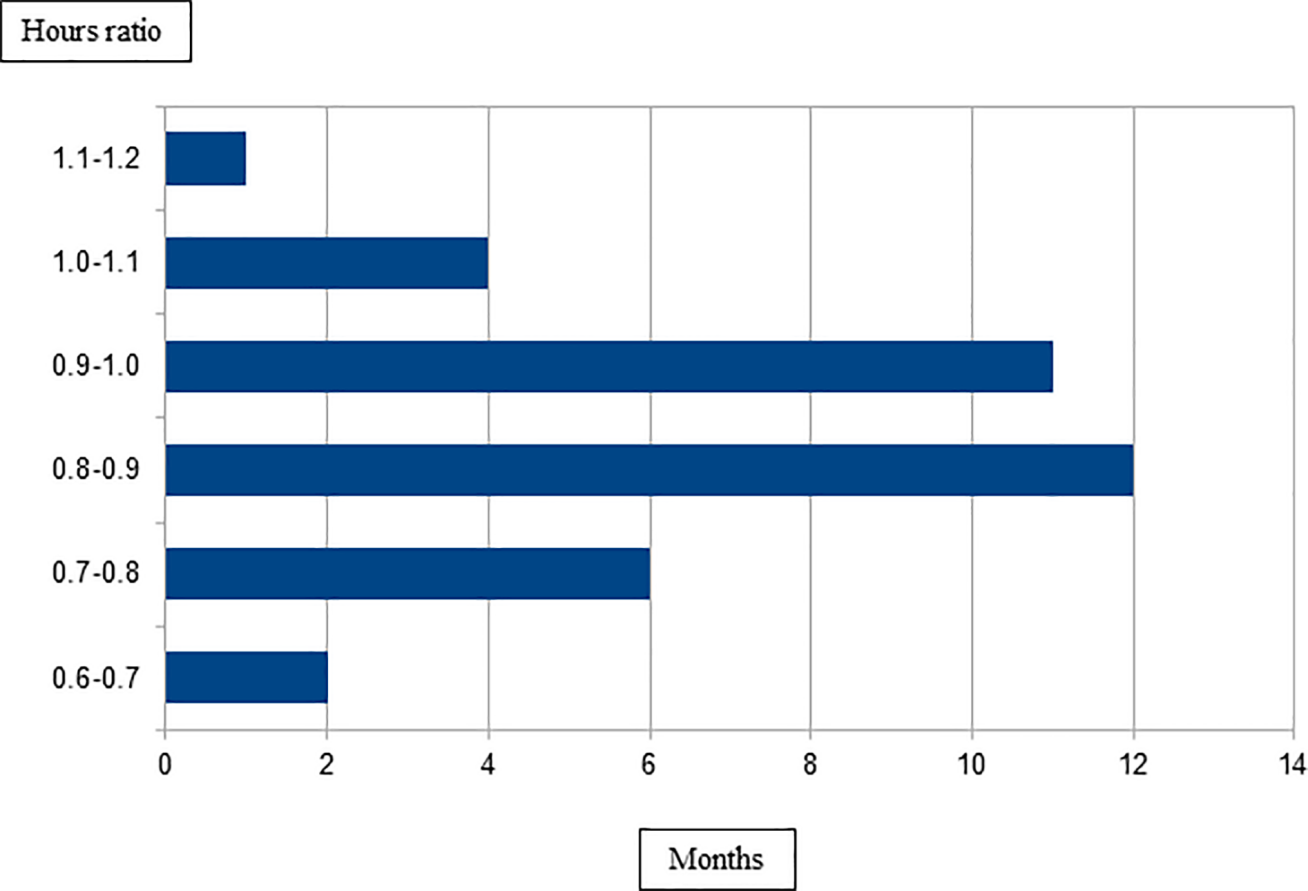
Ratio between the number of nursing care hours provided to the patient and the number of nursing care hours required by the patient. Ribeirão Preto, São Paulo, Brazil, 2013.

Fig 1 illustrates the hours ratio as a function of the number of months. For a period of 12 months, the hours ratio ranged from 0.8 to 0.9; for a period of 11 months, the hours ratio spanned from 0.9 to 1, which is ideal. Nevertheless, for a period of six months, the hours ratio varied from 0.7 to 0.8, which showed that the number of nursing care hours provided to the patient fell well below the required number of nursing care hours. For a period of five months, the hours ratio lay between 1.0 and 1.1, which evidenced that more than the necessary number of nursing care hours was dedicated to the patient.

By correlating the hours ratio with the care indicators according to adjustment models, accidental or unplanned extubation of patients with the orotracheal cannula occurred less frequently when the hours ratio increased.

Analysis of Table 1 showed that pressure ulcer, unplanned removal of the feeding tube, and UTI did not correlate with hours ratio (there was no statistical significance).

**Table 1:** Generalized linear models. Ribeirão Preto, São Paulo, Brazil, 2013.

Model	Coefficient (Razão)		z_value Pr(>|z|)
Model 1 Extot*		-1.6142	-2.2930 0.0296
Model 2 FT†		0.5674	0.5700 0.5729
Model 3 PU‡		-0.9589	-1.2210 0.2310
Model 4 Phlebitis		-9.6670	-2.0910 0.0365
Model 5 UTI§		-1.4715	-0.3860 0.7000
Model 6 VAP||		-13.3040	-2.1050 0.0353

* Accidental or unplanned extubation

† unplanned removal of feeding tube

‡ pressure ulcer

§ urinary tract infection associated with bladder catheter delay

|| ventilation-associated pneumonia.

**Fig 2:**
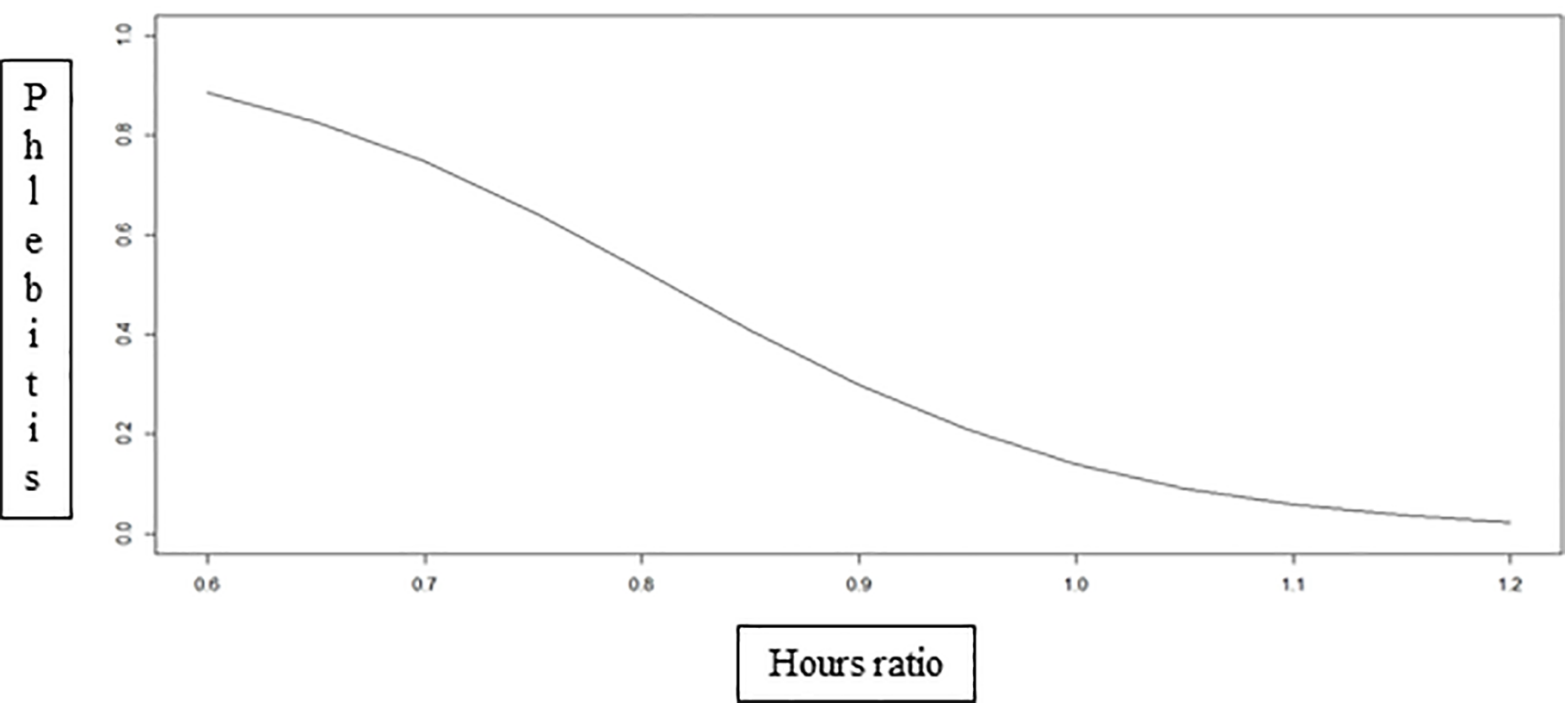
Incidence of phlebitis as a function of the hours ratio. Ribeirão Preto, São Paulo, Brazil, 2013.

**Fig 3:**
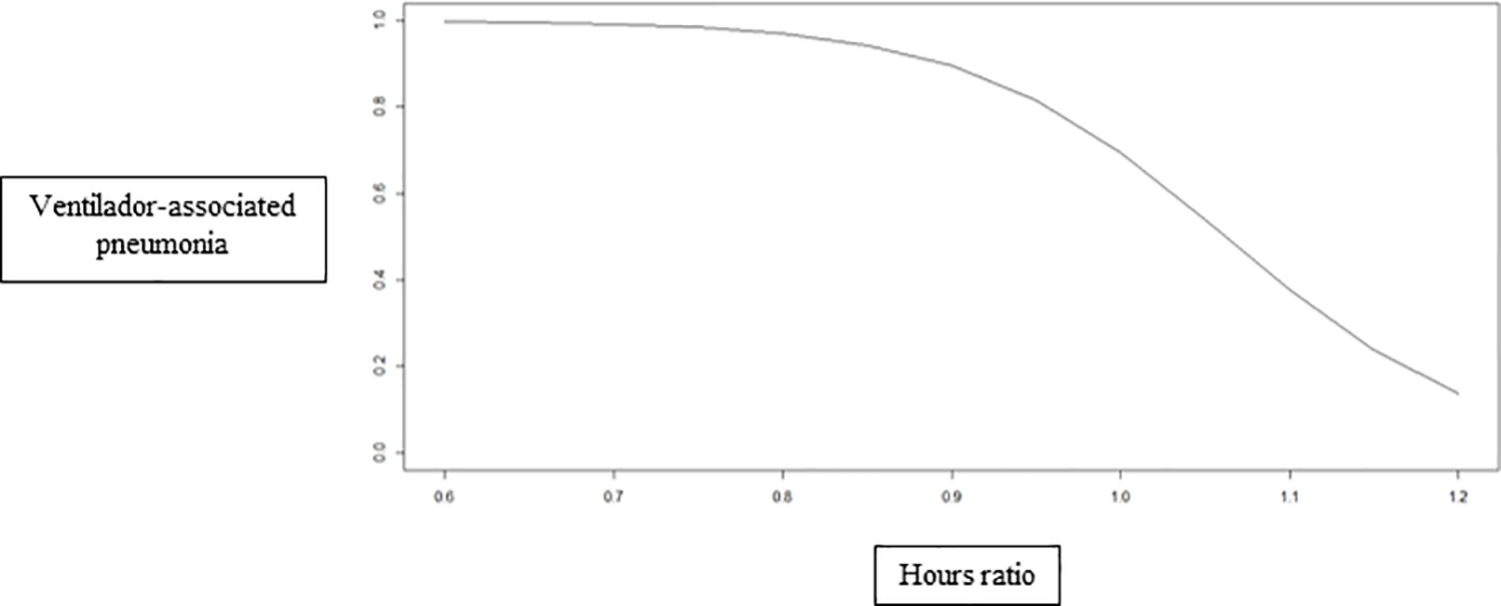
Incidence of ventilation-associated pneumonia as a function of the hours ratio. Ribeirão Preto, São Paulo, Brazil, 2013.

On the basis of the binomial models, higher hours ratio decreased the incidence of phlebitis (Fig 2) and VAP (Fig 3).

## Discussion

An average of 12.21 nursing care hours was provided to the ICU patients along the analyzed period. This value was lower than the values reported in other studies [17,18] and the values recommended by COFEN (Federal Nursing Council)[19] and ANVISA (Brazilian Health Surveillance Agency) [20,21], of 17.9 and 14.4 h in the case of critically ill patients, respectively.

Similarly, the average number of nursing care hours required by the ICU patient, as calculated by the average NAS score, was 54.87% or 13.17 h, which was also lower than the values reported in other investigations [17,18] and the values advocated by COFEN[19] and ANVISA[20,21]. The patients included in this study required less complex care, indicating that the surveyed ICU, which is private, admitted less complex patients given the higher availability of hospital beds and the lower demand for these beds by critically ill patients. The presence of patients classified as semi-intensive in this unit could be related to the lack of specific units to assist this kind of patient, to factors related to the work dynamics of the clinical staff, and to the decision of the medical team to keep semi-intensive patients in the ICU. The presence of less critically ill patients in the ICU incurs higher costs and should not be overlooked by the institution [22].

Regarding the distribution of the average number of nursing care hours provided to the ICU patient based on the professional category (nurse or nurse technician), the number of nursing care hours provided by nurse technicians was higher than the value recommended by COFEN(19) but lower than the value advocated by ANVISA[20,21]. The maximum and the minimum number of nursing care hours provided by nurses was 33% and 19.7%, respectively, which met the provisions of the ANVISA Resolution[21] but not the recommendations of COFEN[19].

A study has evaluated how the nursing staff workload is related to the incidence of adverse events[17]. The study verified that a larger difference between the number of nursing care hours provided to the patient and the number of nursing care hours required by the patient increased the incidence of adverse events. The average incidence of these events was higher in the ICU were nurse staffing was inadequate[17]. Authors investigating an ICU in a university hospital correlated the number of nursing care hours provided to the patient with some indicators of healthcare quality in the context of intensive care. The authors found that the number of nursing care hours correlated only with accidental extubation, and that the incidence of this adverse event decreased when an increasing number of nursing hours was provided to the patient [18].

International studies have shown how the number of nursing care professionals and healthcare indicators, such as mortality and hospital infection, are correlated,[4,8,23–25] but these studies have only considered the number of professionals available at the institution.

The present study has demonstrated that the incidence of accidental or unplanned extubation of the orotracheal cannula, phlebitis, and VAP can be explained in terms of the nursing care hours ratio. The control and surveillance actions provided by the nursing staff to critically ill patients are important and affect patient clinical outcomes. Moreover, the actions undertaken by the nursing staff are relevant to the management of critically ill patients because these actions will help to improve nursing care by considering the number of nursing care hours that can be provided to the patient and the number of nursing care hours that is required by the patient.

This investigation has unique features: sample size, time span, and daily monitoring of patients’ clinical data and demands by a single researcher. Continuous monitoring of healthcare indicators, health surveillance, and assessment of nursing staff procedures allow for earlier intervention and prevent complications in ICU patients.

One limitation of the present study is the use of grouped data. Hence, confounding variables that could affect the incidence of adverse events have not been considered. New studies should target the collection of data by considering patients on an individual basis and adverse events separately, to account for the influence of these factors on patient outcome. Future research could be conducted on the basis of the methodological support presented herein.

## Conclusion

Given that the number of nursing care hours is closely related to patient safety, it is crucial to evaluate the risks that insufficient nurse staffing pose to critical patients.

Here, the use of the ratio between the number of nursing care hours provided to the patient and the number of nursing care hours required by the patient was relevant. The indicators accidental or unplanned extubation of the orotracheal canula, phlebitis, and VAP depended on nurse staffing. The inclusion of indicators to monitor healthcare services is an important strategy to promote patient safety and to negotiate nurse staffing because these indicators evidence how the number of nursing care hours impacts patients and their relatives.

## Supporting information

S1 Table Database: Data of indicators: Incidence of phlebitis, Incidence of unplanned extubation of endotracheal cannula, Ventilator associated pneumonia, Catheter-associated urinary tract infection, Incidence of pressure ulcer, Incidence of unplanned nasogastric / nasoenteric tube for nutritional intake, Nursing Activities Score (monthly average), Hours provided by nursing technicians and Hours provided by nurses. (XLSX)

S2 Table Statistical analysis: Descriptive analysis and Generalized Linear Models. (XLSX)

S3 Table Predicted values: Estimated probability of ventilator-associated pneumonia and phlebitis. (XLSX)
